# Population trends of striped hyena (*Hyaena hyaena*) in Israel for the past five decades

**DOI:** 10.1038/s41598-023-31137-2

**Published:** 2023-03-09

**Authors:** Ezra Hadad, Jakub Z. Kosicki, Reuven Yosef

**Affiliations:** 1 Israel Nature and Parks Authority, 3 Am Ve’Olamo Street, 95463 Jerusalem, Israel; 2 Department of Avian Biology and Ecology, Adam Mickiewicz University, Poznań, Poland; 3 Ben Gurion University of the Negev, Eilat Campus, P. O. Box 272, 8810201 Eilat, Israel

**Keywords:** Population dynamics, Urban ecology, Animal behaviour

## Abstract

The striped hyena (*Hyaena hyaena*) is considered “Near Threatened” globally and “Vulnerable” in the Middle East. In Israel, the species has experienced extreme population fluctuations owing to poisoning campaigns during the British Mandate (1918–1948) which were also further exacerbated by the Israeli authorities in the mid-twentieth century. We collated data from the archives of the Israel Nature and Parks Authority for the past 47 years to elucidate the temporal and geographic trends of this species. During this period we found a 68% increase in population and the estimated density is at present 2.1 individuals/100km^2^. This is significantly higher than all previous estimates for Israel. It appears that the major factors contributing to their phenomenal increase in number are the increase in prey availability because of the intensification of human development, preying on Bedouin livestock, the extinction of the leopard (*Panthera pardus nimr*), and the hunting of wild boars (*Sus scorfa*) and other agricultural pests in some parts of the country. Reasons should also be sought in increasing people's awareness as well as in advanced technological capabilities that have allowed an improved observation and reporting system. Future studies need to understand the effects of the large concentrations of striped hyenas on the spatial distribution and temporal activity of other sympatric wildlife to ensure the continued persistence of the wildlife guilds in the Israeli nature.

## Introduction

The striped hyena (*Hyaena hyaena;* here on hyena) is a large-bodied, facultative nocturnal scavenger^1^. Across most of its range the species occurs mostly in open habitats in arid to semi-arid environments^2^.

The hyena is considered to be globally “Near Threatened” by the International Union for Conservation of Nature (IUCN) Red List, and is reported with a decreasing global population trend^3^. However, regionally for the Middle East it is classified as “Vulnerable”^1^. From the conservation point of view the species is described as one that “experiences ongoing deliberate and incidental persecution coupled with a decrease in its prey base such that it may come close to meeting a continuing decline of 10% over the next three generations^3^.” The main causes of this projection is the abhorrence of this species among humans, including a plethora of superstitious beliefs, e.g., as grave robbers^4^, and/or damage to agriculture and livestock^5–7^. Therefore it is not surprising that various types of systemic and institutional action plans were implemented to reduce hyena population.

In Israel, the species has experienced extremes population declines owing to either targeted or accidental poisonings, and it especially susceptible to this because of their scavenging behavior^8–10^. Historically it was widespread throughout the country^11^ and in the nineteenth century was also very common^12–14^. However, it has been driven to the verge of extinction at the turn of the twentieth century^15,16^. It was the consequence of two temporal and geographically extensive incidents of poising occurred in Israel—the first during the British Mandate when between 1918 and 1948 the authorities placed strychnine poisoned donkey carcasses in order to control Golden Jackals (*Canis aureus*) to try and prevent rabies, but in the process also killed off a large number of hyenas^3^. And the second when Israeli authorities in the 1950s and 1960s assorted to extensive poisoning campaigns, resulting in many species either going regionally extinct or with greatly reduced populations^8,9^.

In the 1970s the estimated population consisted of ca. 130 individuals, i.e., the density for Israel with an area of 22,145 km^2^, was 0.06 hyenas/100 km^2^^17,18^. In the 1990s the estimated population size in the Negev Desert was estimated to be > 0.016/km^2^^19^. At the beginning of the twenty-first century, according to the Israeli Red Book the population size was estimated as ca. 200 individuals, i.e., 0.09/100 km^2^^20^. However, Hadad^21^, estimated the population at ca. 400 individuals (0.18/100 km^2^), but then revised the number to ca. 1000 (0.45/100 km^2^;^16^). Further, the species is persecuted indiscriminately in the West Bank^22,23^, wherein it is killed for its body parts which are considered to have medicinal values, or is killed because of a range of superstitious beliefs connected to the underworld. Hence, the Striped Hyena populations both in Israel and the West Bank in the second half of the twentieth century were reported in relatively low numbers^16,18,21^.

Owing to the uncertainty of the status of the hyenas in Israel, and the different levels of human and ecological pressures that they experience across the length of the country, and based on a 47-year time series data, we developed a temporal and geographic model of the population trend of the species in Israel. We hypothesized that the termination of targeted persecution by the authorities in the first half of the twentieth century and the rapid urbanization of central and northern Israel would be conducive to reviving local hyena populations. Allowing that this is now the largest carnivore in the country that interacts frequently with humans, it is of conservation concern that the human attitudes towards this large carnivore remain positive.

## Methods

### Data collection

**Figure 1:**
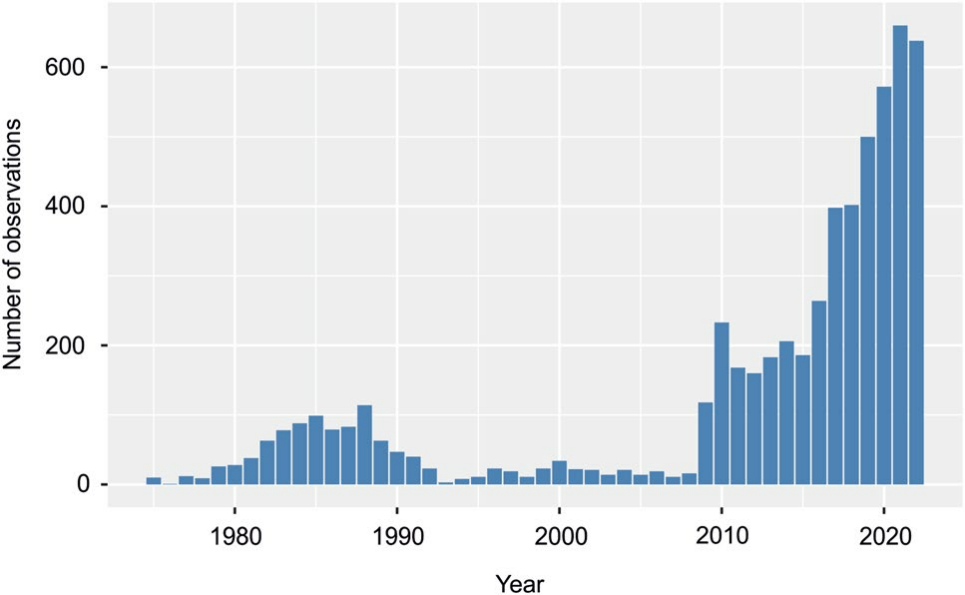
Raw numbers of observations per year of striped hyena (*Hyaena hyaena*) in Israel.

The data for estimating the population trend of the hyena in Israel and the West Bank were obtained from the Scientific Data Department of the Israel Nature and Parks Authority (INPA). The INPA was created in 1998 by merging two independent organizations—The National Parks Authority and The Nature Reserves Authority (NRA; https://en.parks.org.il/). The NRA was established in 1964 as the official arm of the Israeli Government to enforce wildlife protection laws. During the years 1975—2022, i.e., a total of 47 years, the INPA has detailed records of 5,860 observations of hyena (Fig. 1), which we have used in the present analyses. Initially, i.e., during 1975–2008, the observations recorded were those submitted by INPA rangers and citizens. From 2008, the INPA introduced the application from smartphone “Cyber Ranger” to report observation directly from the field.

### Data processing

**Figure 2:**
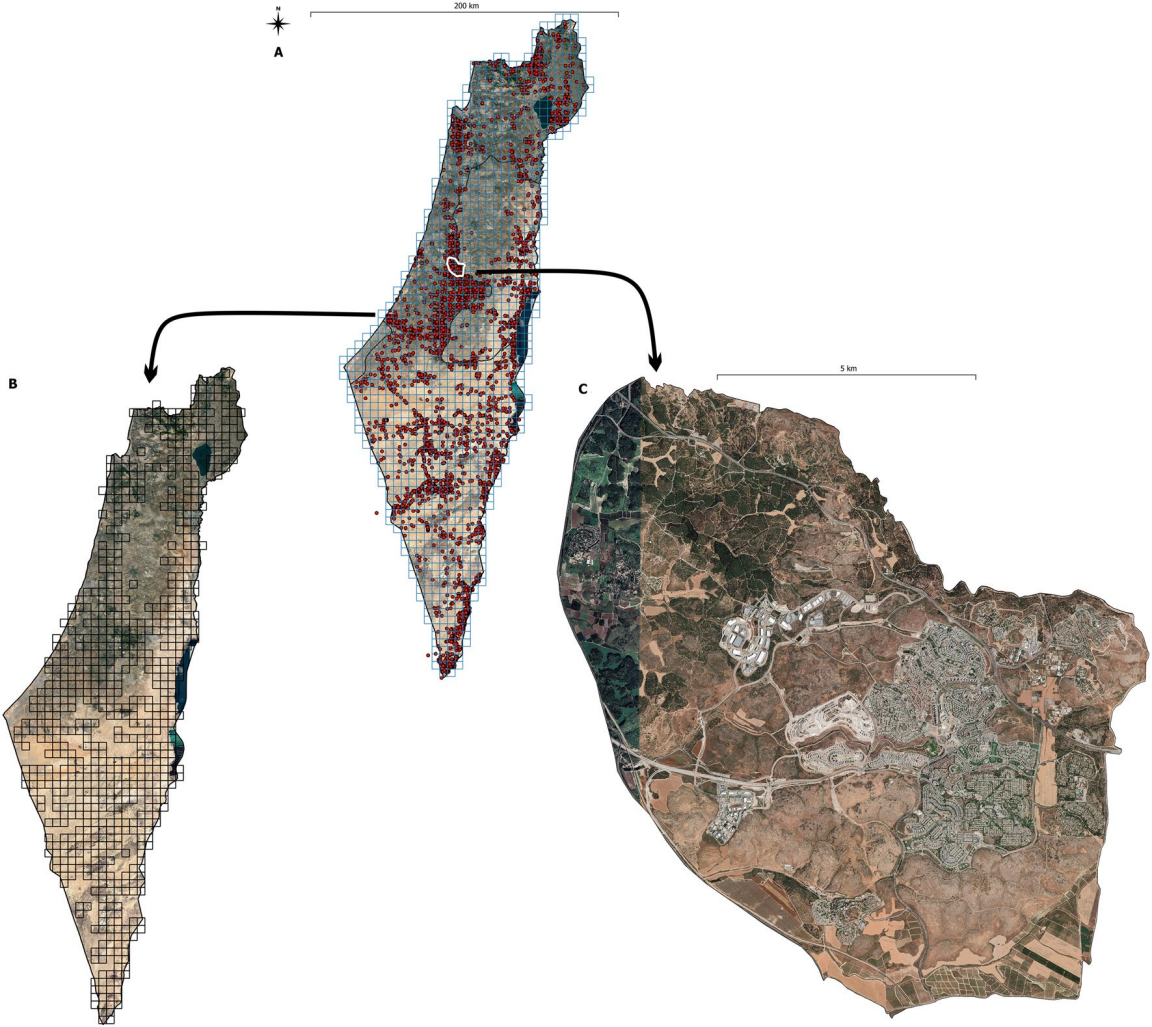
Map of Israel and the West Bank (**A**) with red dots marking the locations of the striped hyena (*Hyaena hyaena*) over 47 years of research, and 1255 analytical squares (blue); (**B**) analytical squares in which they were found at least once in 47 years; (**C**) the research area in which the density was precisely determined. The background is from Google Earth.

All observations were assigned geographical coordinates (Fig. 2A). However, the hyenas are not individually marked, and we were unable to control for the effect of reporting the same individual multiple times in a year. Thus, in order to balance the concentration of observations from urbanized areas with open areas (with low human activity) and at the same time to minimize the probability of population overestimation, we combined observations into time-spatial groups.

In the first step, we divided Israel and the West Bank into 5/5 km squares (Fig. 2A). As a result, we got 1255 analytical surfaces (the coordinates of the center of each square are shown in Online Appendix B). Next, separately for each of the 47 years, we assigned each observation to a given analytical surface assuming that two or more adults reported within a year and at a distance of not more than 1000 m, are sightings of the same individual (Fig. 2B). Using this approach, we believe that we have to some degree controlled the effect of randomness of observation, which is common in citizen science collected data.

### Statistical analysis

To evaluate the trend analysis we used generalized additive mixed models GAMM where the number of individuals in the given grid cells (*y*_*gr*_) was considered as an additive effect of year and analytical surface (grid cells). In the modeling framework we used ‘year’ as the fixed factor (*f(year)*) and the id of grid cells as the random factor. The model was developed with Poisson distribution. The analysis was based on the *ptrend* procedure implemented in the *poptrend* library (for theoretical background of the analysis see Knape^24^).

We also used generalized additive mixed models GAMM^25^ to analyze not only in time (temporal) but also in space (geographical) population trends. Namely, the annual rate of population change was modeled as a function of the latitude and longitude of a given grid cell. In this way, apart from determining the population trend, we were able to indicate on a specific map for the variability of the population trend (positive/negative) in the whole of Israel. As the response variable we used the logarithm of the number of individuals of a given grid cell “g” in year “r”. This variable was modeled with a Poisson-Gamma distribution (pg). The ‘Year’ variable was added to the modeling framework as a fixed factor (f(year)) and as a continuous variable in interactive relation with the longitude and latitude (s(longitude, latitude, year)). Furthermore, the id of the grid cells (id_gc) was used as the random factor. The model was considered in two variants: spline *s* and linear *l;* such that the equation was:

$$\begin{gathered} {\text{y}}_{{{\text{gr}}}} \sim {\text{ p}}_{{\text{g}}} \left( {{\text{a}}_{{{\text{gr}}}} \sigma^{{2}} } \right),{\text{ a}}_{{{\text{gr}}}} \sim {\text{ log}}\left( {{\text{a}}_{{{\text{gr}}}} } \right) \, = \, \phi_{{{\text{gr}}}} \hfill \\ \phi_{{{\text{gr}}}} = {\text{ f}}\left( {{\text{year}}} \right) \, + {\text{ s}}\left( {{\text{longitude}},{\text{ latitude}}} \right) \, + {\text{ s}}\left( {{\text{longitude}},{\text{ latitude}},{\text{ year}}} \right) \, + {\text{ id}}\_{\text{gc}} \\ \& \\ \phi_{{{\text{gr}}}} = {\text{ f}}\left( {{\text{year}}} \right) \, + {\text{ l}}\left( {{\text{longitude}},{\text{ latitude}}} \right) \, + {\text{ l}}\left( {{\text{longitude}},{\text{ latitude}},{\text{ year}}} \right) \, + {\text{ id}}\_{\text{gc}} \hfill \\ \end{gathered}$$

where *y*_*gr*_*—o*f number of individuals of a given grid cells g in year r, *pg—*Poisson-Gamma distribution, s—spline, *gc*—id of grid cell.

The differences between the two models were tested using likelihood ratio tests.

Furthermore, we also calculated annual population growth rate (λ) which is an exponential model and ensures the estimate of statistical growth of the population per year^26^.

$$n_{y2} = \, n_{y1} \times \, \lambda^{(y2 - y1)}$$

where *n*_*y2*_*—*number of individuals in year 2, while *n*_*y1*_ means number of individuals in the year preceding *n*_*y2.*_

Based on the next GAMM we also estimated the species density. Response variable were expressed as the quotient of the population size in a given grid cell in year and the population trend obtained from the model described above. This variable was implemented in GAMM with Poisson-Gamma distribution where as predictor interactive and additive effect longitude and latitude and year were used. Based on this model we created predictive maps of the spatial density distribution.

### Institutional review board statement

The study did not require ethical approval and was conducted in the framework of the Israel Nature & Parks Authority.

### Ethics

The data is from the archives of the Scientific Data Department of the Israel Nature and Parks Authority, and is used under their jurisdiction. No animals were handled in the study.

## Results

During the 47-year study, we recorded a total of 5,860 individuals in 723 grid cells out of 1255 that cover all of Israel and the West Bank. The mean value of individual per year was 131.2 (95% CL 77.63–184.99) while, per grid cell was 8.71 (95% CL 4.41–10.02). The fewest individuals were observed in 1976 and 1995 (1 and 3, respectively), while the most individuals were recorded in 2022 (703). We found a linear increasing trend in the number of reported individuals over the past 47 years (r = 0.68, p < 0.001, Fig. 1). All observations were assigned geographical coordinates (Fig. 2A) and illustrated that most observations were near urban agglomerations.

**Figure 3:**
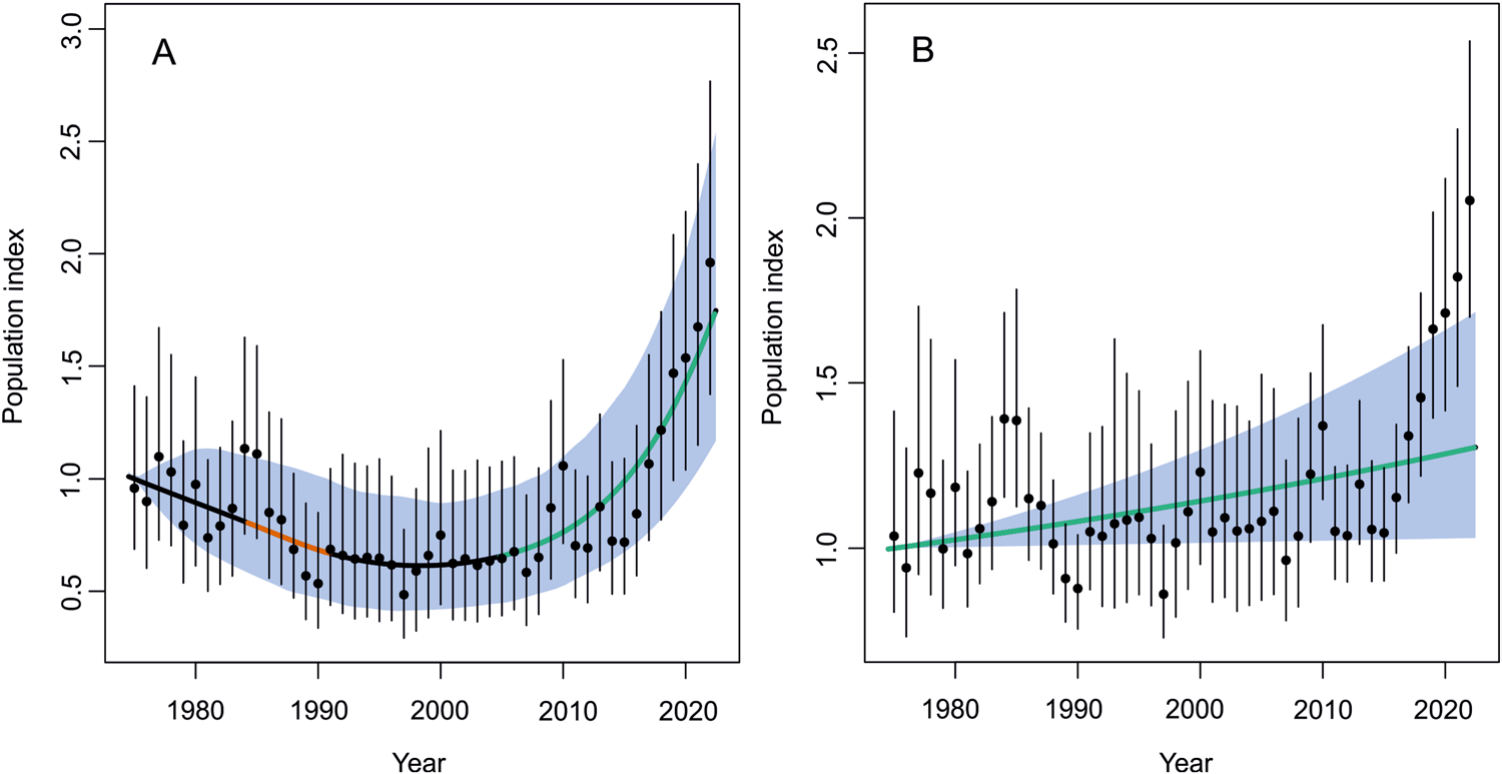
Population trends of the striped hyena (*Hyaena hyaena*) in Israel and the West Bank based on GAMM with smooth (**A**) and loglinear fits (**B**). Error bars show the 95% confidence limits around the yearly estimates; black, red and green lines show smooth-fitted to the yearly point estimates, blue area represents 95% CL for the smooth fit.

**Figure 4:**
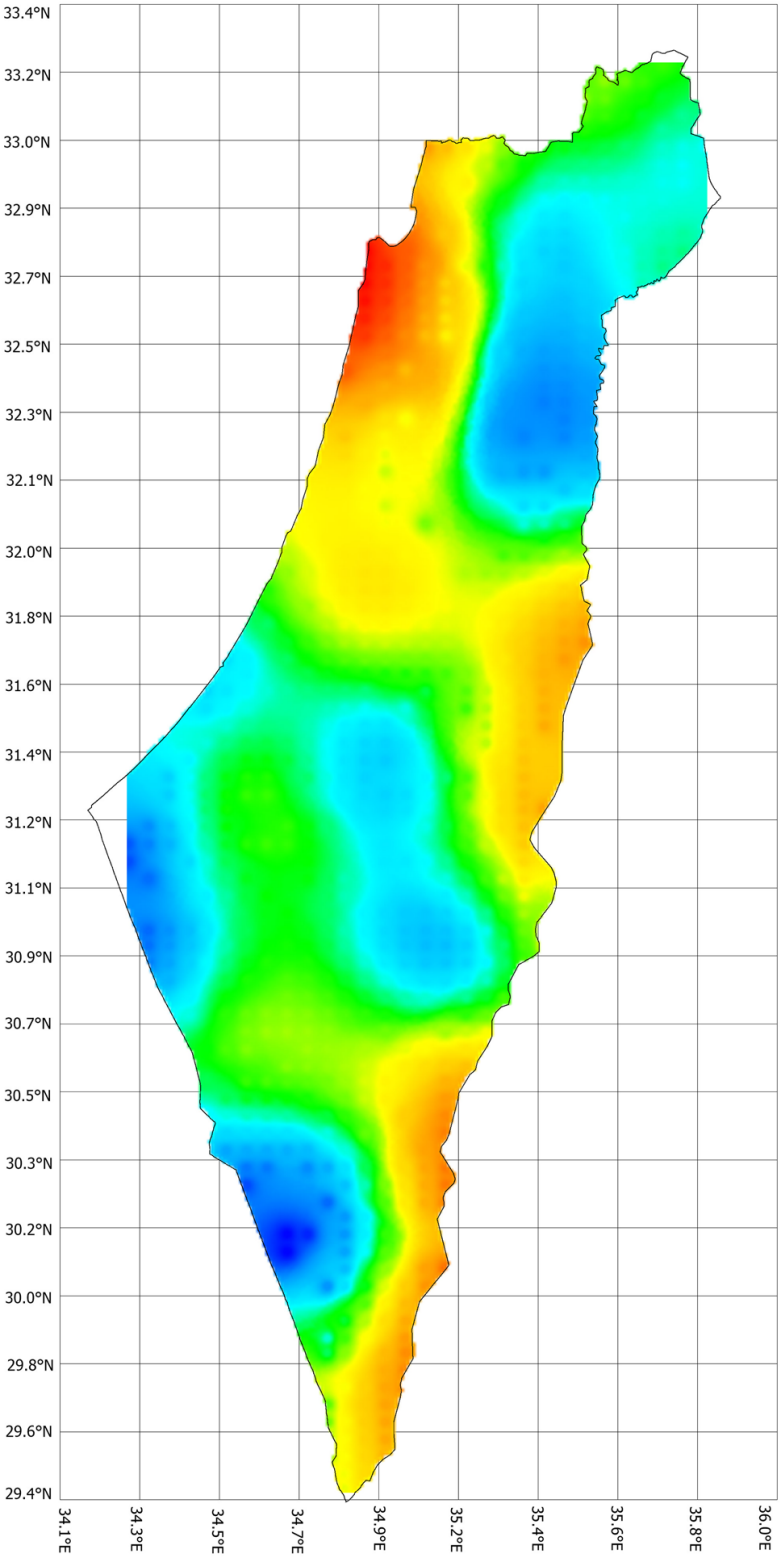
Time-spatial variability of the population trend of striped hyena in Israel, 1975–2022. Blue areas indicate population growth ≤ 10%, while red areas indicate population growth ≥  90%.

Trend population modeling based on GAMM with "smooth" fit (R^2^ = 0.294) showed that in Israel between 1975 and 2022 the hyena population increased on average by 68% (95% CL 10–161%). However, on a scale of 47 years, we identified a period of population decline between 1984 and 1990, in which the population decreased by − 16% (95% CL − 27 to − 3.8%) and in 2005–2022 the population increased by 156% (95% CL 87–245%; Figs. 3A, 4). The loglinear fit (Fig. 3B) also showed an increased population trend, but the model with the "smooth" fit was better (LSR test, chi-square = 34.76, P < 0.0001) than the linear. The annual population growth rate (λ) in the exponential model for the entire period = 1.53 (95% CL 0.96–2.10), indicating that on average the population increase by 53% per year.

**Figure 5:**
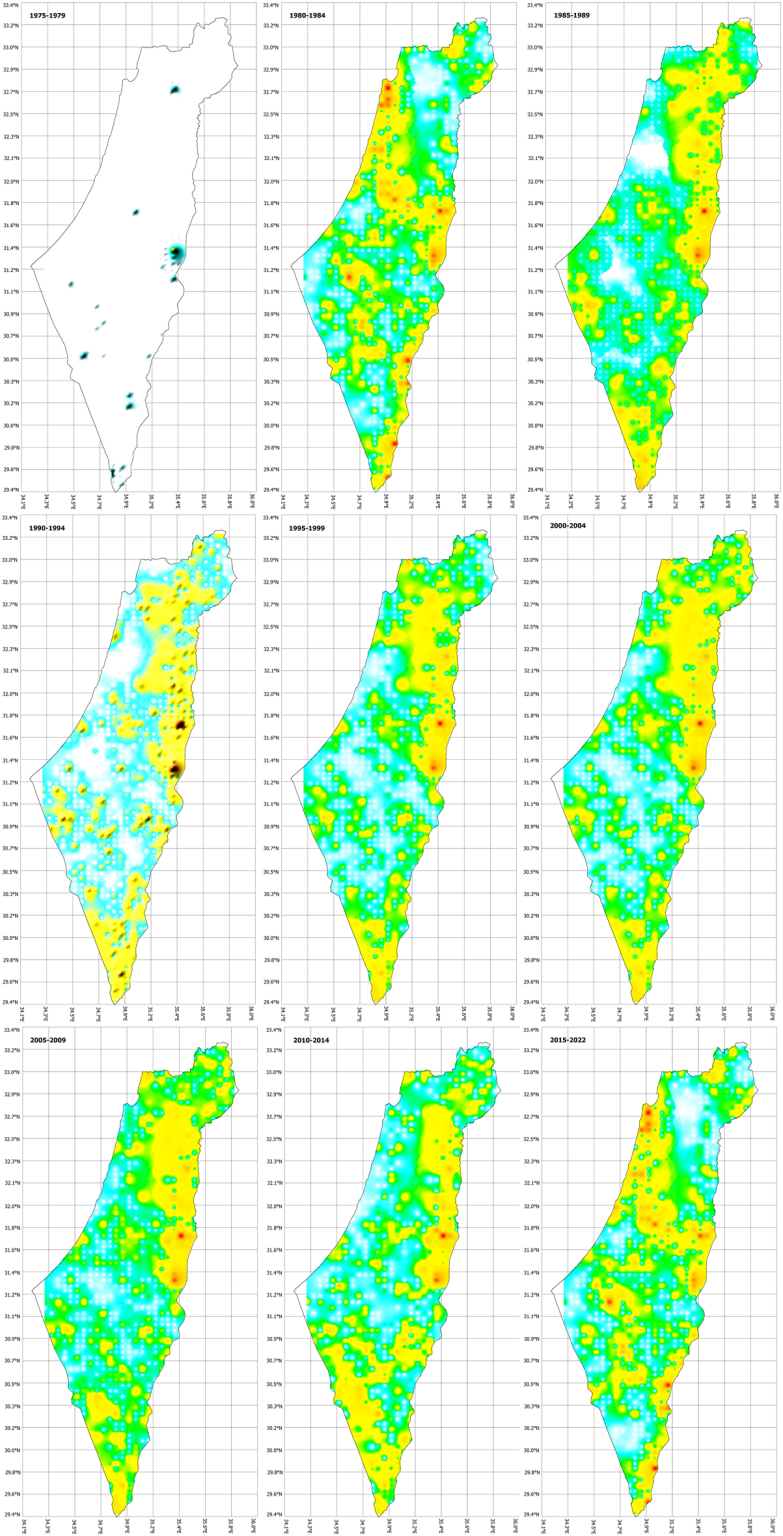
The changes in the density of the striped hyena (*Hyaena hyaena*) in Israel and the West Bank during nine sequential periods between 1975 and 2022.

The estimated density for the whole country is currently 2.1 individuals/100km^2^ (95% CL 0.3–6.2), but we found a large spatial variance (Fig. 5). However, the predicted density in the controlled study area (Fig. 2C) was 0.22 per/km^2^ (95% CL 0.20–0.25) and was slightly higher than the density for the whole country and lower than the real density found during field studies in this area (0.34 individuals/km^2^). However, the differences between real and predicted density were not statistically significant (chi-square = 0.018, p = 0.89).

## Discussion

The hyena, despite being elusive, cryptic, nocturnal, and solitary^1,27–29^ and mainly found in semi-arid and arid regions^18,30^; in Israel, appears to have overcome most environmental restrictions and at present occupies all habitats^31,32^. They also appear to have overcome their fear of humans and have become human commensals^33–35^. We discovered that most observations were near urban agglomerations. We assume that this may be due to two reasons—first, increased hyena activity in specific areas owing to access to anthropogenic food base^33^. Extensive areas for human habitation and agriculture is accompanied by and large quantities of garbage dumps, resulting in not only the commensal carnivore populations increasing greatly^36^ but also an increase in body size, which was also reported for hyenas^33^. Furthermore, establishing of feeding stations (restaurants) for scavengers has allowed access to food for carnivores, including hyenas^37^. The second reason for the high activity of hyenas near urban agglomerations may be due from a methodological artifact where higher human activity in reporting observed animals in urban areas than in open areas.

Ilani^17^ reported that in Israel hyenas were either non-existent or extremely rare for a 10–25 km wide area along the Mediterranean coastline from Rafiah in the Gaza strip to Rosh Hanikra on the border with Lebanon; and also inland in the Jezreel Valley and the lower Galilee regions. The species also existed in low numbers in the Upper Galilee and the Golan Heights, and from the Gilboa Mountains and south along the rift valley, the numbers were small but stable.

In most Middle East countries, the status of the hyena ranges from “Vulnerable” to “Critically Endangered”^4,38–41^, including “Endangered” in Israel^20^. However, one should be very careful in changing this status to higher, e.g., based on the population growth trends we obtained. The population numbers are mainly dependent on habitat fragmentation of optimal habitats^40,41^ and human persecution in the form of poisoning^4,8,9^, illegal wildlife trade^23,28^, retaliatory killing^39^, collection of sexual organs for traditional medicines^42^, and a result from the disproportionately large rate of mortality as road kills^10,28,42^. Climate change also appears to influence hyena populations, but the influence on the Israeli population remains unknown^43^.

So the only solutionis similar to Ilani^17^, a country-wide monitoring survey to elucidate the actual regional densities and reassess the species status and how the authorities can ensure the continued persistence of a viable population in Israel. It is necessary because, despite our use of a complex analytical procedure and independent evaluation of our estimation, there is still the risk of under- or overestimating the population size. This study is based on archival data, albeit correct to the present because it is based on observations and not regular surveys. The assumption that two or more hyena sightings within a year at a distance of < 1000 m belong to the same individual may be potentially misleading given that hyenas exhibit a complex social structure^44^. However, this approach is the only solution to ensure that the population is estimated correctly. We came to this conclusion during a heuristic review of the data. The points lying < 1000 m apart is the supposed area of a single individual. Furthermore, as described before, the degree of interaction between individuals^44^ despite all may vary between seasons, something that has yet to be described for this species. Thus, no clear evidence exists that hyena sightings < 1000 m away belong to two or more individuals.

Next, the assignment post-hoc of observations to the analytical square and the predictive procedure also can generate random distortions that cannot be avoided in this kind of citizen science, especially since the data comes from a period of almost 50 years. Over the years, there has been a change in people's awareness. Advanced technological capabilities have allowed an improved observation (e.g., smartphone applications) and reporting system that has dramatically increased the number of reports filed. However, this also resulted in the same individuals being reported over the years with no ability to discern between them.

However, no matter the level of underestimation, there is no doubt that the hyena population is increasing in Israel. This is indirectly ground-proofed in the number of road-kills^10^ and other studies in specific regions^16,18,19,21,30–32^. The extensive fieldwork and ground-proofing of almost 4-decades supports this conclusion in the Judea region in central Israel^10,15,16,21^.

As mentioned earlier, the benign behavior of the hyenas and the fact that they do not endanger humans has resulted in some regions they are now tolerated to the point where they have become human commensals^33–35^. We suspect, this has also led to a lower perception of risk by the hyena, but it requires further study. We speculate that the rapid population growth is the consequence of intensification of human development—whether as the building of new towns and cities and expanding old ones, introducing new roads and infrastructure, and increasing the acreage of agricultural and silvicultural areas^45^. Furthermore, agricultural and industrial intensification produces much organic waste in the desert regions, which the hyenas exploit. Also, in the semi-arid regions, where the Bedouins allow their livestock to forage in the winters, this results in an abundance of domestic prey readily available for the hyenas^46^. The extinction of the leopard (*Panthera pardus nimr*)^47–49^ essentially leaves the hyena as the largest of the Carnivora in the country with minimal intra-specific competition^53^.

Similar to Shamon et al.^32^, we also found the highest density of hyenas in the Carmel Mountains in northern Israel. We assume this is probably a result of the increase in prey availability of “agricultural pests” in the region and the subsequent human-wildlife conflict^50^. Hunters were contracted to kill as many wild boars (*Sus scorfa*) and golden Jackal (*Canis aureus*) as possible and whose carcasses were not removed^16^. This rich resource allowed foraging carnivores, including hyenas, to establish substantial populations within a relatively small area quickly. However, unlike Panda et al.^51^ we are unable to elucidate the competitive inter- or intra-specific interactions with other carnivores.

Our results show that Israel’s hyena density is 2.1 individuals/100 km^2^. In central Kenya, the estimated minimum density was 0.3 adults/100 km^2^^2^, while estimates for neighboring Lebanon were 0.11/100 km^2^^4^. Several studies have also been conducted on striped hyena densities in central India (0.45/100 km^2^;^52^) and Gir National Park in Gujarat in western India (0.7/100 km^2^;^53^). However, the highest density was observed in Kumbhakgarh (6.5/100 km^2^) and at Esrana (3.7/100 km^2^;^54^), as well as at Ranthambore Tiger Reserve (5.5/100 km^2^), and the Sawai Mansingh Wildlife Sanctuary (12.0/100 km^2^;^35^).

In conclusion, we present the most recent evaluation of the hyena population in Israel and the West Bank and demonstrate that the species appears to have recovered dramatically from previous poisoning episodes, despite it being persecuted in certain regions by specific ethnic groups^6,23,27,38,55^, that it has rebounded impressively^6,38,40^. The hyenas have learned to exploit human waste and are not afraid of humans. However, the coexistence between the wild hyena population and humans must be treated with caution^56^. Their proximity to human habitation and lack of fear of humans can result in undesired conflicts in the future, similar to that occurring in the Mount Carmel region with wild boars^50^. The relevant authorities must indulge in education programs explaining the dangers of contact with wildlife and feeding them to ensure that public health is not susceptible and that the hyena populations are monitored for expansion and human exploitation. The authorities must try to nullify the anthropogenic influence on the hyena’s high densities and evaluate the environment’s actual carrying capacity for this large carnivore. The ramifications of these studies can also be projected to other areas of the species distribution^57^ and help the species persist in the wild across its range.

## Supplementary Information

Supplementary Information.

## Data Availability

The data is available directly from the author, Reuven Yosef, ryosef60@gmail.com.
